# The Osteosarcoma Stem Cell Activity of a Gallium(III)‐Phenanthroline Complex Appended to Salicylate

**DOI:** 10.1002/cbic.202200532

**Published:** 2022-11-18

**Authors:** Ruby A. Vincent, Ginevra Passeri, Joshua Northcote‐Smith, Kuldip Singh, Kogularamanan Suntharalingam

**Affiliations:** ^1^ School of Chemistry, University of Leicester LE1 7RH Leicester UK

**Keywords:** antitumor agents, bioinorganic chemistry, gallium, non-steroidal anti-inflammatory drugs, osteosarcoma stem cells

## Abstract

We report the synthesis, characterisation, and anti‐osteosarcoma properties of a gallium(III) complex (**1**) comprising of two 1,10‐phenanthroline ligands and salicylate, a non‐steroidal anti‐inflammatory drug. The gallium(III) complex **1** displays micromolar potency towards bulk osteosarcoma cells and osteosarcoma stem cells (OSCs). Notably, the gallium(III) complex **1** exhibits significantly higher toxicity towards OSCs grown in monolayer and three‐dimensional cultures than cisplatin, a frontline anti‐osteosarcoma drug. Nuclei isolation and immunoblotting studies show that the gallium(III) complex **1** enters osteosarcoma cell nuclei and induces DNA damage. Flow cytometry and cytotoxicity studies (in the presence of prostaglandin E2) indicate that the gallium(III) complex **1** downregulates cyclooxygenase‐2 (COX‐2) expression and kills osteosarcoma cells in a COX‐2‐dependent manner. Further, the mode of osteosarcoma cell death evoked by the gallium(III) complex **1** is characterised as caspase‐dependent apoptosis.

## Graphical abstract

**Osteosarcoma** is the most commonly diagnosed primary bone cancer and has strikingly low survival rates. Here we report the anti‐osteosarcoma properties of a six‐coordinate gallium(III) complex with two polypridyl ligands and salicylate, a non‐steroidal anti‐inflammatory drug. The gallium(III) complex kills bulk osteosarcoma cells and osteosarcoma stem cells in the micromolar range by evoking apoptosis through DNA damage and cyclooxygenase‐2 downregulation.

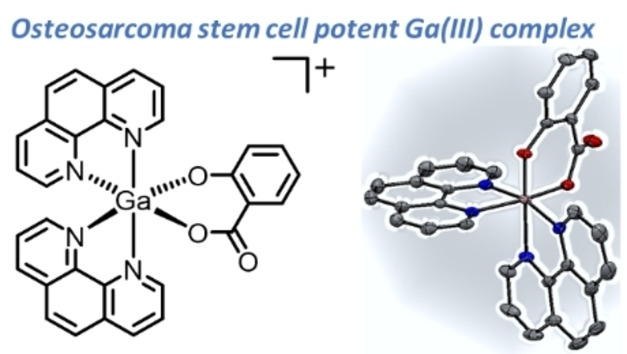

## Introduction

Osteosarcoma is the most common primary cancer of the bone and is defined by the presence of malignant mesenchymal cells, which produce osteoid and immature bone.^[1]^ Osteosarcoma arises in bones during periods of rapid growth, and for this reason, it is thought to be common in adolescence due to growth spurts associated to puberty (it is the third most common cancer in adolescents).^[2]^ The standard care for patients presenting with osteosarcoma includes (neo)adjuvant chemotherapy, surgery, and sometimes radiation.^[3]^ Frontline chemotherapeutic regimens include high‐dose methotrexate with leucovorin rescue, cisplatin, and doxorubicin.^[4]^ These frontline treatment options have not changed for several decades. Second‐line chemotherapeutic options include ifosfamide, etoposide, and carboplatin, however, the ideal combination of both frontline and second‐line drugs remains to be defined.^[5]^ A major shortcoming associated to the current array of anti‐osteosarcoma chemotherapeutics is systemic toxicity, a consequence of their non‐selectivity for osteosarcoma over healthy tissue.^[2]^ Another pitfall is their inability to completely prevent relapse and spread to secondary sites (metastasis).^[6]^ Osteosarcoma stem cells (OSCs), a drug resistant sub‐population of self‐renewing osteosarcoma cells are thought to be responsible for osteosarcoma progression, recurrence, and metastasis.^[7]^ Given the poor prognosis of metastasised osteosarcoma (5‐year survival rate is only 20 %) and the link to OSCs,^[8]^ the development of chemotherapeutics that can remove both bulk osteosarcoma cells and OSCs could be vital for improving long term clinical outcomes for osteosarcoma patients.

Reports on the development of chemotherapeutics that can remove OSCs (at clinically relevant doses) are extremely rare, and have mainly focused on purely organic compounds.^[9]^ We recently reported the first metal‐based agents to potently kill OSCs grown in monolayer and sarcosphere (three‐dimensional) cultures.^[10]^ A family of cationic gallium(III) compounds containing two bidentate polypyridyl ligands and two chloride ligands were found to kill bulk osteosarcoma cells and OSCs within the micro‐ to nano‐molar range.^[10]^ The most effective gallium(III) compound in this series was over 400‐fold more potent than cisplatin towards methotrexate‐resistant OSCs, and significantly less toxic towards a panel of non‐cancerous cells of various tissue types (lung, breast, skin, and kidney).^[10]^ Following this, we reported a ‘second generation’ gallium(III) compound containing two bidentate polypyridyl ligands and diflunisal, a non‐steroidal anti‐inflammatory drug (NSAID), with improved anti‐OSC properties.^[11]^ The gallium(III) compound displayed significantly higher monolayer and sarcosphere OSC potency (up to three orders of magnitude) than clinically approved drugs used in frontline (doxorubicin and cisplatin) and second‐line (etoposide, ifosfamide, and carboplatin) osteosarcoma treatments.^[11]^ Advanced cell‐based studies showed that the gallium(III) compound induced osteosarcoma cell death by damaging nuclear DNA and downregulating cyclooxygenase‐2 (COX‐2) expression.^[11]^ Here, we have sought to expand this promising class of gallium‐polypyridyl‐NSAID anti‐OSC agents by incorporating salicylate (the principal metabolite of aspirin). Aspirin has been shown to suppress both the growth and metastasis of osteosarcoma at the cellular level and in animal models.^[12]^ Specifically, herein we report the synthesis and characterisation of a novel gallium(III)‐polypyridyl complex appended to salicylate and provide insight into the anti‐OSC potential and mechanism of action of the complex.

## Results and Discussion

### Synthesis and characterisation

**Figure 1:**
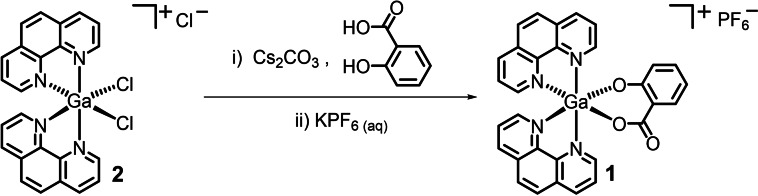
Reaction scheme for the preparation of the gallium(III)‐polypyridyl complex bearing salicylate **1** from *cis*‐dichlorobis‐(1,10‐phenanthroline) gallium(III) chloride complex **2**.

**Figure 2:**
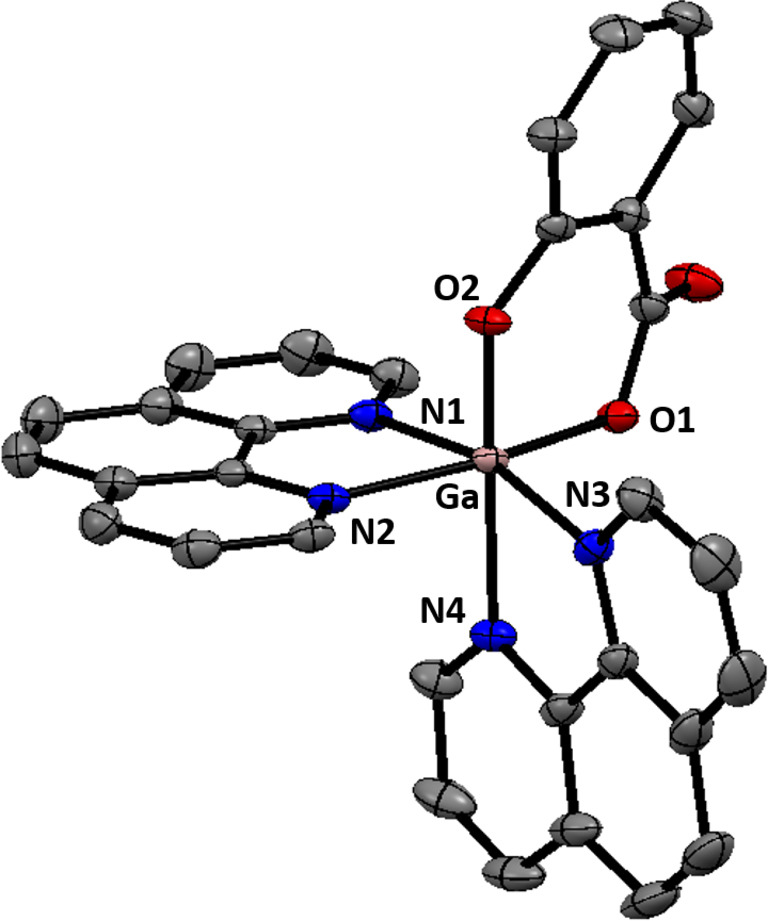
X‐ray structure of **1** comprising of two 1,10‐phenanthroline ligands and a salicylate moiety. The hexafluorophosphate counter‐anion and the co‐crystallizing solvent molecule (acetonitrile) have been omitted for clarity. Ellipsoids are shown at 50 % probability. N atoms are shown dark blue, C in grey, O in red, and Ga in pink.

The gallium(III) complexes investigated in this study are depicted in Figure 1. The gallium(III)‐polypyridyl complex containing salicylate **1** was prepared by reacting *cis*‐dichlorobis‐(1,10‐phenanthroline) gallium(III) chloride **2** with equimolar amounts of salicylic acid in methanol, in the presence of CsCO_3_. The *cis*‐dichlorobis‐(1,10‐phenanthroline) gallium(III) chloride complex **2** was prepared using a previously reported protocol.^[13]^ After reaction of **2** with salicylic acid and subsequent conversion to the corresponding hexafluorophosphate salt (using potassium hexafluorophosphate), the crude product was dried in dichloromethane with Na_2_SO_4_, and recrystallized using acetonitrile and diethyl ether to give pure **1** as an off‐white solid in a reasonable yield (39 %). The gallium(III) complex **1** was fully characterised by ^1^H, ^31^P{^1^H}, ^19^F{^1^H} NMR, infrared spectroscopy, high resolution ESI mass spectrometry, and elemental analysis (Figures S1–S5). Within the ^1^H NMR spectrum of **1**, the aromatic proton signals shifted relative to salicylic acid, indicative of metal coordination (Figure S6). The fact that only one set of ^1^H NMR signals were observed for the salicylate moiety in **1** suggests that it binds to the gallium(III) centre via only one orientation. The IR spectrum for **1** displayed ν_asym_(CO_2_) and ν_sym_(CO_2_) stretching bands at 1631 cm^−1^ and 1430 cm^−1^, respectively (Figure S4). The difference, Δ, between the ν_asym_(CO_2_) and ν_sym_(CO_2_) stretching bands for **1** was 201 cm^−1^, suggestive of an unidentate coordination mode for the carboxylate group (on the salicylate moiety) to the gallium(III) centre.^[14]^ A distinctive molecular ion peak corresponding to **1** with the appropriate isotopic pattern was observed in the positive mode of the high resolution ESI mass spectrum (*m/z*=565.0790 amu, [**1**‐PF_6_]^+^), providing further evidence for product formation (Figure S5). Crystals of **1** suitable for X‐ray diffraction analysis were obtained by slow diffusion of diethyl ether into an acetonitrile solution of **1** (CCDC 2205005, Figure 2, Table S1). Selected bond distances and bond angles are presented in Table S2. The cationic component of the complex exhibits a distorted octahedral geometry with the gallium(III) centre coordinated to two 1,10‐phenanthroline ligands and a salicylate moiety via two hydroxyl groups. The gallium(III) coordination sphere is consistent with the aforementioned spectroscopic and analytic data for **1**. The average Ga−N (2.10 Å) and Ga−O (1.89 Å) bond distances are consistent with bond parameters for related gallium(III) complexes.[^11^, ^15^]

### Lipophilicity and solution stability

The lipophilicity of the gallium(III) complexes **1** and **2** was experimentally determined by measuring the extent to which they partitioned between octanol and water, using UV‐Vis spectroscopy. The LogP value for **1** was 0.27±0.01, noticeably higher than the LogP value for **2** which was −0.48±0.01 (Table S3). The difference in LogP values for **1** and **2** is expected as **1** contains a hydrophobic salicylate moiety. The LogP value for **1** indicates that the gallium(III) complex should be able to readily enter cells and have reasonable aqueous solubility.

UV‐Vis spectroscopy studies were performed to determine the stability of the salicylate‐containing gallium(III) complex **1** in various solutions relevant for subsequent cell‐based studies. In DMSO, H_2_O:DMSO (200 : 1), and PBS:DMSO (200 : 1) the absorbance and wavelengths of the bands associated to **1** (50 μM) remained largely unaltered over the course of 24 h at 37 °C, indicative of stability (Figures S7–S9). To probe the solution stability of **1** further, the gallium complex (50 μM) was incubated with 10 equivalents of ascorbic acid (a cellular reductant) in PBS:DMSO (200 : 1) and monitored by UV‐Vis spectroscopy for 24 h at 37 °C. The UV‐Vis trace for **1** was only very slightly modified under these conditions, suggestive of good stability (Figure S10). Before carrying out cell‐based studies, the stability of **1** in dye‐free (unsupplemented) Dulbecco's Modified Eagle Medium (DMEM) was investigated (Figure S11). The UV‐Vis trace of **1** (50 μM) in DMEM:DMSO (200 : 1) displayed no changes over the course of 24 h at 37 °C. Therefore **1** was deemed suitably stable to progress to cell‐based studies.

### Osteosarcoma potency in monolayer and three‐dimensional cultures

The cytotoxicity of the salicylate‐containing gallium(III) complex **1** towards bulk osteosarcoma cells (U2OS) and OSC‐enriched cells (U2OS‐MTX) grown in monolayers was assessed using the MTT [3‐(4,5‐dimethylthiazol‐2‐yl)‐2,5‐diphenyltetrazolium bromide] assay. IC_50_ values (concentration required to reduce cell viability by 50 %) were interpolated from dose‐response curves (Figure S12) and are summarised in Table 1. The gallium(III) complex **1** exhibited micromolar potency towards U2OS and U2OS‐MTX cells. The potency of the *cis*‐dichlorobis‐(1,10‐phenanthroline) gallium(III) chloride complex **2** towards U2OS and U2OS‐MTX cells was similar to **1** (Figure S13, Table 1), implying that the addition of the salicylate moiety to the *cis*‐bis‐(1,10‐phenanthroline) gallium(III) unit in **1** does not adversely affect its osteosarcoma cell potency or spectrum of activity. The toxicity of **1** towards OSC‐enriched U2OS‐MTX cells was 4‐fold (*p*<0.05, n=18) greater than cisplatin, a frontline anti‐osteosarcoma drug.^[10]^ The gallium(III) complex **1** was however less toxic than doxorubicin (another clinically used frontline anti‐osteosarcoma drug) and salinomycin (a clinically tested agent with strong activity against stem cell‐like tumour sub‐populations) towards U2OS‐MTX cells.[^10^, ^11^] Control cytotoxicity studies showed that salicylic acid was non‐toxic (IC_50_ value >100 μM) towards U2OS and U2OS‐MTX cells (Figure S14, Table 1). In light of the toxicity data for **2** and salicylic acid (Table 1) and the similarity in potency of **1** and **2** towards U2OS and U2OS‐MTX cells, it appears that the *cis*‐bis‐(1,10‐phenanthroline) gallium(III) unit within **1** is most likely responsible for the bulk osteosarcoma cell and OSC toxicity observed for **1**. Of note, the gallium(III) complexes **1** and **2**, and cisplatin killed U2OS cells preferably over U2OS‐MTX cells while doxorubicin killed U2OS and U2OS‐MTX cells somewhat equally (Table 1). This suggests that doxorubicin (and not **1**, **2** or cisplatin) could potentially remove whole osteosarcoma populations comprising of bulk osteosarcoma cells and OSCs with a single dose. Further, **1** was markedly less potent towards U2OS and U2OS‐MTX cells than analogous gallium(III)‐polypyridyl complexes containing diflunisal.^[11]^ This implies that the nature of the NSAID present influences the osteosarcoma cell potency of gallium(III)‐polypyridyl‐NSAID complexes.

**Table 1:** IC_50_ values of the salicylate‐containing gallium(III) complex **1**, *cis*‐dichlorobis‐(1,10‐phenanthroline) gallium(III) chloride complex **2**, salicylic acid, doxorubicin, cisplatin, and salinomycin against U2OS and U2OS‐MTX cells and sarcospheres.

Compound	U2OS IC_50_ [μM]^[a]^	U2OS‐MTX IC_50_ [μM]^[a]^	Sarcosphere IC_50_ [μM]^[b]^
**1**	1.31±0.03	7.96±1.14	2.60±0.01
**2**	1.10±0.13	8.64±0.40	2.77±0.02
salicylic acid	>100	>100	>133
cisplatin^[c]^	16.30±0.50	33.87±3.71	16.49±0.20
doxorubicin^[c]^	0.17±0.01	0.22±0.07	0.19±0.08
salinomycin^[c]^	6.09±1.06	1.49±0.26	4.70±0.08

[a] Determined after 72 h incubation (mean of three independent experiments±SD). [b] Determined after 10 days incubation (mean of two independent experiments±SD). [c] Reported in references [10] and [11].

Additional monolayer cytotoxicity studies to assess the potency of **1** towards non‐cancerous epithelial breast MCF10A cells were performed. The gallium(III) complex **1** was significantly less potent towards MCF10A cells (IC_50_=3.04±0.06 μM, *p*<0.05, n=18, Figure S15) than U2OS cells. Contrastingly, **1** was significantly more potent towards MCF10A cells than U2OS‐MTX cells.

**Figure 3:**
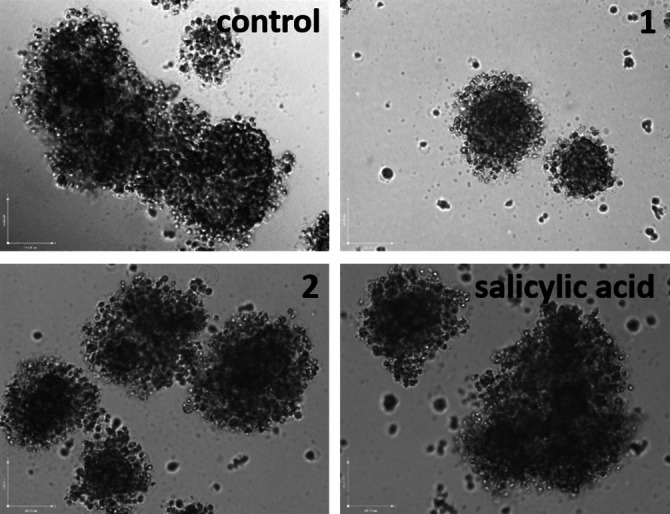
Representative bright‐field images (×10) of U2OS‐MTX sarcospheres in the absence and presence of **1**, **2** or salicylic acid at their respective IC_20_ values for 10 days.

Given the promising activity of the salicylate‐containing gallium(III) complex **1** towards OSCs cultured in monolayers, we tested its ability to impact OSCs grown in three‐dimensional cultures (using the sarcosphere assay). Sarcospheres are free‐floating spheroidal structures made up of OSCs.^[16]^ Sarcospheres provide a good model for gauging OSC potency and *in vivo* potential, and can be generated in serum‐free, low attachment conditions. Dosage of single cell suspensions of U2OS‐MTX cells with **1** (at the IC_20_ value for 10 days) markedly reduced the size of sarcospheres formed (Figure 3). The addition of **2**, doxorubicin, and salinomycin had a similar or better inhibitory effect on sarcosphere formation than **1** (when treated at their respective IC_20_ values for 10 days) (Figures 3 and S16). Under identical conditions, cisplatin and salicylic acid did not noticeably affect sarcosphere size (Figures 3 and S16). Considering the contrasting sarcosphere inhibitory effects of **2** and salicylic acid, it is implicative that the *cis*‐bis‐(1,10‐phenanthroline) gallium(III) moiety within **1** is a determining factor in the sarcosphere inhibitory effect of **1**. To establish the effect of **1** on sarcosphere viability, TOX8 a resazurin‐based reagent was employed. The IC_50_ values (concentration required to reduce sarcosphere viability by 50 %) were interpolated from dose‐response curves (Figure S17) and are summarised in Table 1. The gallium(III) complex **1** displayed micromolar toxicity towards sarcospheres, comparable to **2** and salinomycin and 6‐fold (*p*<0.05, n=18) greater than cisplatin (Figure S17, Table 1).^[10]^ The gallium(III) complex **1** was less potent than doxorubicin towards sarcospheres (Table 1). Based on the IC_50_ values, **1** was 3‐fold more potent towards three‐dimensionally cultured sarcospheres than U2OS‐MTX cells grown in monolayer cultures (Table 1). This is an extraordinary result given that most small molecules exhibit lower potency towards spheroidal systems compared to monolayer systems (made up of the same cells). As expected, salicylic acid was non‐toxic (IC_50_ value >133 μM) towards sarcospheres (Figure S17, Table 1). Taken together, the sarcosphere studies suggests that **1** is able to effectively inhibit the formation and reduce the viability of OSC‐enriched sarcospheres.

### Mechanism of cytotoxicity: DNA damage, cyclooxygenase‐2 downregulation, and apoptosis

**Figure 4:**
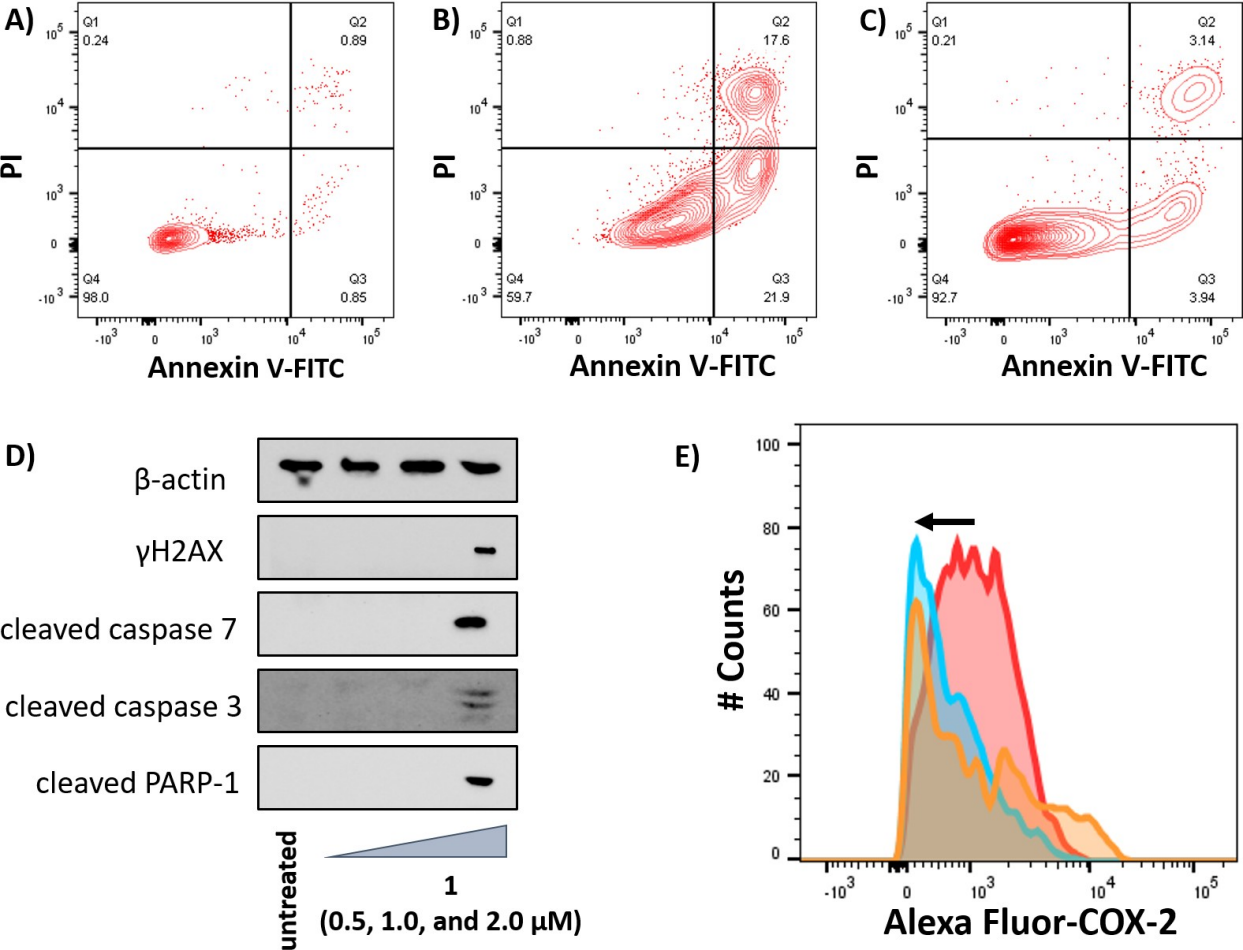
FITC Annexin V‐propidium iodide binding assay plots of (A) untreated U2OS cells, (B) U2OS cells treated with **1** (IC_50_ value×2 for 72 h), and (C) U2OS cells treated with cisplatin (12.5 μM for 72 h). (D) Immunoblotting analysis of proteins related to the DNA damage response and apoptosis pathways. Protein expression in U2OS cells following treatment with **1** (0.5, 1.0, and 2.0 μM) for 48 h. (E) Representative histograms displaying the green fluorescence emitted by anti‐COX‐2 Alexa Fluor 488 nm antibody‐stained U2OS‐MTX cells treated with LPS (2.5 μg/mL) for 24 h followed by 48 h in fresh media (red) or media containing **1** (IC_50_ value, blue) or **1** (IC_50_ value×2, orange).

Our previous work on a structurally related gallium(III)‐polypyridyl compound containing diflunisal (an NSAID akin to salicylate) showed that it induced osteosarcoma cell apoptosis by damaging genomic DNA and downregulating COX‐2 expression.^[11]^ Therefore, we first investigated the ability of 1 to enter the nuclei in osteosarcoma cells and induce genomic DNA damage. Nuclei isolation studies found that when U2OS cells were incubated with **1** (5 μM) for 24 h at 37 °C, a substantial amount of internalised **1** (4.91±0.35 ng of Ga/10^6^ cells) was detected in the nuclei (16 %, 0.77±0.03 ng of Ga/10^6^ cells). Given that **1** is able to access osteosarcoma cell nuclei it is possible that **1**‐mediated osteosarcoma cell toxicity could be associated to genomic DNA damage. The ability of **1** to activate a specific biomarker related to the DNA damage response pathway was probed using immunoblotting studies. U2OS cells dosed with **1** (0.5–2.0 μM for 48 h) displayed a marked increase in the expression of the phosphorylated form of H2AX (γH2AX) (Figure 4D). Accumulation of γH2AX results from direct phosphorylation of the histone H2A variant, H2AX, by ATM or ATR at sites of DNA lesions.^[17]^ Therefore, the increase in γH2AX levels observed upon treatment of U2OS cells with **1** suggests that **1** induces DNA damage in osteosarcoma cells. Given that **1** is able to enter the nuclei in osteosarcoma cells to a significant level and upregulate a pertinent DNA damage marker (γH2AX) within 24–48 h (Figure 4D), we believe that DNA damage is a primary effect of **1**.

DNA damage can translate into cell death via apoptosis if left unrepaired.^[18]^ Apoptosis prompts structural changes that can lead to cell membrane rearrangement. As part of this process, phosphatidylserine residues are translocated to the membrane exterior, which can be detected by Annexin V.^[19]^ Damaged cell membranes also facilitate propidium iodide uptake. Using a dual FITC Annexin V‐propidium iodide staining flow cytometry assay, we explored the occurrence of apoptosis in U2OS cells treated with **1**. Dosage with **1** (IC_50_ value×2 for 72 h) induced large populations of U2OS cells to undergo early‐ and late‐stage apoptosis relative to untreated control cells (Figures 4A–B). Appreciable populations of U2OS cells treated with cisplatin (12.5 μM for 72 h) also appeared to undergo early‐ and late‐stage apoptosis but this was to a significantly lesser extent than upon treatment with **1** (Figure 4C). According to immunoblotting studies, U2OS cells treated with **1** (0.5–2.0 μM for 48 h) exhibited higher levels of cleaved caspase 3, 7, and poly ADP ribose polymerase (PARP) compared to untreated cells (Figure 4D), characteristic of caspase‐dependent apoptosis. To further corroborate caspase‐dependent apoptosis, cytotoxicity studies were carried out in the presence of z‐VAD‐FMK (5 μM, 72 h), a peptide‐based caspase‐dependent apoptosis inhibitor.^[20]^ The IC_50_ value of **1** towards U2OS‐MTX cells increased significantly in the presence of z‐VAD‐FMK (IC_50_ value=14.17±2.13 μM, *p*<0.05, Figure S18) further confirming that **1** induces caspase‐dependent apoptosis in osteosarcoma cells. Collectively the nuclei isolation, immunoblotting, flow cytometry, and cytotoxicity studies indicate that **1** can access osteosarcoma cell nuclei, inflict genomic DNA damage, and trigger caspase‐dependent apoptosis.

As the gallium(III) complex **1** contains a salicylate moiety, which is known to inhibit and downregulate COX‐2,^[21]^ we investigated whether **1** induced COX‐2 dependent osteosarcoma cell death. U2OS‐MTX cells pre‐treated with lipopolysaccharide (LPS) (2.5 μg/mL for 24 h), to increase basal COX‐2 levels, were treated with **1** (IC_50_ value and IC_50_ value×2 for 48 h) and the COX‐2 expression was determined by flow cytometry. A marked decrease in COX‐2 expression was observed for U2OS‐MTX cells treated with **1** (Figure 4E) compared to untreated cells. This suggests that the cytotoxic mechanism of action of **1** may involve COX‐2 downregulation. U2OS‐MTX cells treated with salicylic acid (20 μM for 48 h) also showed an appreciable decrease in COX‐2 expression (Figure S19). To further prove that **1** evokes COX‐2‐dependent osteosarcoma cell death, cytotoxicity studies were performed with U2OS‐MTX cells in the presence of prostaglandin E2 (PGE2) (20 μM, 72 h), the functional product of COX‐2‐mediated arachidonic acid metabolism.^[22]^ The potency of **1** towards U2OS‐MTX cells decreased significantly in the presence of PGE2 (IC_50_ value=13.50±0.07, *p*<0.05) (Figure S20), suggesting that **1** induces osteosarcoma cell death through a COX‐2‐dependent pathway. Overall, the COX‐2 associated flow cytometry and cytotoxicity studies confirmed that **1** induces osteosarcoma cell death via a COX‐2‐dependent pathway.

## Conclusion

In summary we report the preparation, characterisation, and osteosarcoma cell activity of a six‐coordinate gallium(III) complex (**1**) containing two 1,10‐phenanthroline ligands and a salicylate moiety. The X‐ray crystal structure of **1** revealed a distorted octahedral structure with the salicylate moiety bound to the gallium(III) centre via two hydroxyl groups. The gallium(III) complex **1** was stable in physiologically relevant solutions (water, PBS, and cell culture media) and in the presence of excess amounts of ascorbic acid. Cytotoxicity studies showed that **1** killed bulk osteosarcoma cells and OSCs, grown in monolayers, in the micromolar range. Notably **1** was more potent towards bulk osteosarcoma cells and OSCs than cisplatin, but less active than doxorubicin and salinomycin. The gallium(III) complex **1** was also significantly less potent towards bulk osteosarcoma cells and OSCs than analogous gallium(III)‐polypyridyl complexes containing diflunisal (as opposed to salicylate). This suggests that the nature of the NSAID present has a determining role in the osteosarcoma cell potency of gallium(III)‐polypyridyl‐NSAID complexes. The gallium(III) complex **1** was able to disturb three‐dimensionally cultured sarcosphere formation to a similar extent as salinomycin, and to a better extent than cisplatin. Interestingly, **1** was significantly more potent towards three‐dimensionally cultured sarcospheres than OSCs cultured in monolayers. This is an unusual characteristic for small molecules and suggests that **1** is able to penetrate the multicellular sarcosphere structure, and thus could be highly translatable (with respect to *in vivo* studies). Furthermore, **1** was significantly more potent towards sarcospheres than cisplatin, comparable to salinomycin, and less potent than doxorubicin. Mechanistic experiments involving nuclei isolation, immunoblotting, flow cytometry, and cytotoxicity studies (in the presence of various signalling pathway inhibitors) suggest that **1** induces osteosarcoma cell apoptosis by accessing osteosarcoma nuclei, damaging genomic DNA, and downregulating COX‐2 expression. Overall, this study showcases the therapeutic potential of gallium(III)‐polypyridyl compounds containing NSAIDs, particularly as potential drug candidates for osteosarcoma treatments.

## Experimental Section

**Materials and methods**: All synthetic procedures were performed under normal atmospheric conditions. Mass spectra were recorded on a Micromass Quattro with the electrospray (ESI) technique and on a Kratos Concept 1H (ESI‐TOF). UV‐Vis absorption spectra were recorded on a Cary 3500 UV‐Vis spectrophotometer. ^1^H, ^31^P{^1^H} and ^19^F{^1^H} NMR were recorded at room temperature on a Bruker Avance 400 spectrometer (^1^H 400.0 MHz, ^31^P 162.0 MHz, ^19^F 376.5 MHz) with chemical shifts (δ, ppm) reported relative to the solvent peaks of the deuterated solvent. Fourier transform infrared (FTIR) spectra were recorded with an IRAffinity‐1S Shimadzu spectrophotometer. Inductively coupled plasma mass spectrometry (ICP‐MS) were measured using a Thermo Scientific iCAP−Qc quadrupole ICP mass spectrometer. Elemental Analysis was performed commercially at the University of Cambridge. Salicylic acid, Cs_2_CO_3_, and KPF_6_ were purchased from Sigma Aldrich or Alfa Aesar and used as received. Solvents were purchased from Fisher and used without further purification. The *cis*‐dichlorobis‐(1,10‐phenanthroline) gallium(III) chloride complex **2** was prepared according to a previously reported procedure.^[13]^

**Synthesis of [Ga^III^(1,10‐phenanthroline)_2_(salicylate)](PF_6_), 1**: A solution of salicylic acid (26.5 mg, 0.19 mmol) and Cs_2_CO_3_ (63.6 mg, 0.19 mmol) in MeOH (8 mL) was added to a solution of *cis*‐dichlorobis‐(1,10‐phenanthroline) gallium(III) chloride complex **2** (102.0 mg, 0.19 mmol) in MeOH (20 mL). The reaction mixture was stirred at ambient temperature under an aerobic atmosphere for 20 h. A precipitate crashed out during the reaction, which was removed by filtration. The solvent was removed, and the resultant solid was dissolved in MeOH (5 mL) and stirred with KPF_6_ (320.0 mg, 1.74 mmol) in MeOH (2 mL) for 10 min. Distilled water (40 mL) was added and a precipitate formed. The precipitate was collected by filtration and washed with H_2_O (30 mL×2) and Et_2_O (20 mL). The resultant solid was dissolved in DCM (20 mL) and dried with Na_2_SO_4_ (5 g) for 20 min. Upon removal of Na_2_SO_4_ by filtration and DCM under vacuum, the resulting solid was dissolved in the minimum amount of acetonitrile and Et_2_O was added. The resulting precipitate was collected by filtration and dried under vacuum to yield **1** as an off‐white solid (52.6 mg, 39 %); ^1^H NMR (400 MHz, CD_3_CN): δH 9.50 (1H, dd), 9.42 (dd, 1H), 9.12 (d, 1H), 9.10 (d, 1H), 8.82–8.76 (m, 2H), 8.40–8.34 (m, 3H), 8.32–8.26 (m, 3H), 7.94 (dd, 1H), 7.88 (dd, 1H), 7.86 (dd, 1H), 7.77–7.70 (m, 2H), 7.22 (ddd, 1H), 6.68 (ddd, 1H), 6.58 (dd, 1H); ^31^P{^1^H} NMR (162 MHz, CD_3_CN): δP −144.64 (sept); ^19^F{^1^H} NMR (376 MHz, CD_3_CN): δF −72.93 (d); ATR‐FTIR (solid, cm^−1^): 1631, 1589, 1523, 1468, 1454, 1430, 1332, 1229, 1136, 1108, 1037, 835, 765, 740, 721, 668, 649, 584, 556, 427; ESI‐MS Calcd. for C_31_H_20_N_4_O_3_Ga [M−PF_6_]^+^: 565.0791 a. m. u. Found [M−PF_6_]^+^: 565.0790 a. m. u.; Anal. Calcd. for C_31_H_20_N_4_O_3_F_6_PGa ⋅ 1.75H_2_O (%): C, 50.13; H, 3.19; N, 7.54. Found: C, 49.79; H, 2.74; N, 7.29.

**X‐ray single crystal diffraction analysis**: Single crystals of complex **1** were obtained by slow diffusion of diethyl ether into an acetonitrile solution of **1**. Crystals suitable for X‐ray diffraction analysis were selected and mounted on a Bruker Apex 2000 CCD area detector diffractometer using standard procedures. Data was collected using graphite‐monochromated Mo−Kα radiation (λ=0.71073) at 150(2) K. Crystal structures were solved and refined using the Bruker SHELXTL software.^[23]^ All hydrogen atoms were located by geometrical calculations, and all non‐hydrogen atoms were refined anisotropically. Deposition Number 2205005 contains the supplementary crystallographic data for this paper. These data are provided free of charge by the joint Cambridge Crystallographic Data Centre and Fachinformationszentrum Karlsruhe Access Structures service www.ccdc.cam.ac.uk/structures.

**Measurement of water‐octanol partition coefficient (LogP)**: The LogP values for **1** and **2** were determined using the shake‐flask method and UV‐Vis spectroscopy. The 1‐octanol used in this experiment was pre‐saturated with water. An aqueous solution of **1** and **2** (500 μL, 100 μM) was incubated with 1‐octanol (500 μL) in a 1.5 mL tube. The tube was shaken at room temperature for 24 h. The two phases were separated by centrifugation and the **1** and **2** content in each phase was determined by UV‐Vis spectroscopy.

**Cell lines and cell culture conditions**: The U2OS bone osteosarcoma and the human epithelial breast MCF10A cell lines were acquired from American Type Culture Collection (ATCC, Manassas, VA, USA). U2OS cells were cultured in Dulbecco's Modified Eagle's Medium (DMEM) supplemented with 10 % foetal bovine serum and 1 % penicillin. MCF10A cells were maintained in Mammary Epithelial Cell Growth Medium (MEGM) with supplements and growth factors (BPE, hydrocortisone, hEGF, insulin, and gentamicin/amphotericin‐B). The cells were grown at 37 °C in a humidified atmosphere containing 5 % CO_2_. To gain access to OSC‐enriched cells, a full T75 flask of U2OS cells was treated with methotrexate (300 nM) for 4 days. The cells (labelled U2OS‐MTX cells) were then used immediately. U2OS‐MTX cells were characterised according to CD117 expression using flow cytometry as previously reported.[^10^, ^16^]

**Cytotoxicity MTT assay**: The colorimetric MTT assay was used to determine the toxicity of **1**, **2**, and salicylic acid. U2OS, U2OS‐MTX or MCF10A cells (5×10^3^) were seeded in each well of a 96‐well plate. After incubating the cells overnight, various concentrations of the compounds (0.0004–100 μM) were added and incubated for 72 h (total volume 200 μL). Stock solutions of the compounds were prepared as 10 mM solutions in DMSO and diluted using media. The final concentration of DMSO in each well was ≤0.5 % and this amount was present in the untreated control as well. After 72 h, 20 μL of a 4 mg/mL solution of MTT in PBS was added to each well, and the plate was incubated for an additional 4 h. The DMEM/MTT or MEGM/MTT mixture was aspirated and 200 μL of DMSO was added to dissolve the resulting purple formazan crystals. The absorbance of the solutions in each well was read at 550 nm. Absorbance values were normalized to (DMSO‐containing) control wells and plotted as concentration of test compound versus % cell viability. IC_50_ values were interpolated from the resulting dose dependent curves. The reported IC_50_ values are the average of three independent experiments, each consisting of six replicates per concentration level (overall n=18).

**Sarcosphere formation and viability assay**: U2OS‐MTX cells (5×10^3^) were plated in ultralow‐attachment 96‐well plates (Corning) and incubated in DMEM supplemented with N2 (Invitrogen), human EGF (10 ng/mL), and human bFGF (10 ng/mL) for 10 days. Studies were also conducted in the presence of **1**, **2**, salicylic acid, cisplatin, doxorubicin, and salinomycin (0–133 μM). Sarcospheres treated with **1**, **2**, salicylic acid, cisplatin, doxorubicin, and salinomycin (at their respective IC_20_ values, 10 days) were imaged using an inverted microscope. The viability of the sarcospheres was determined by addition of a resazurin‐based reagent, TOX8 (Sigma). After incubation for 16 h, the fluorescence of the solutions was read at 590 nm (λ_ex_=560 nm). Viable sarcospheres reduce the amount of the oxidized TOX8 form (blue) and concurrently increases the amount of the fluorescent TOX8 intermediate (red), indicating the degree of sarcosphere cytotoxicity caused by the test compound. Fluorescence values were normalized to DMSO‐containing controls and plotted as concentration of test compound versus % sarcosphere viability. IC_50_ values were interpolated from the resulting dose dependent curves. The reported IC_50_ values are the average of two independent experiments, each consisting of three replicates per concentration level (overall n=6).

**Cellular accumulation and nuclei isolation studies**: To measure the cellular accumulation of **1**, *ca*. 1 million U2OS cells were treated with **1** (5 μM) at 37 °C for 24 h. After incubation, the media was removed and the cells were washed with PBS (2 mL×3), and harvested. The number of cells was counted at this stage, using a haemocytometer. This mitigates any cell death induced by **1** at the administered concentration and experimental cell loss. The cells were centrifuged (1500 rpm for 5 min) to form a pellet. The cellular pellet was dissolved in 65 % HNO_3_ (250 μL) overnight to determine whole cell accumulation. The cellular pellet was also used to determine the gallium content in the nuclei. The Thermo Scientific NE‐PER Nuclear and Cytoplasmic Extraction Kit was used to extract and separate the nuclear fraction. The nuclear fraction was dissolved in 65 % HNO_3_ overnight (250 μL final volume). All samples were diluted 17‐fold with water and analysed using ICP‐MS (Thermo Scientific iCAP−Qc quadrupole). Both ^69^Ga and ^71^Ga isotopes were measured using the He mode. Gallium levels are expressed as Ga (ng) per million cells. Results are presented as the mean of four determinations for each data point.

**Immunoblotting analysis**: U2OS cells (5×10^3^ cells) were incubated with **1** (0.5, 1.0, and 2.0 μM) for 48 h at 37 °C. Cells were washed with PBS, scraped into SDS‐PAGE loading buffer (64 mM Tris‐HCl (pH 6.8)/9.6 % glycerol/2 % SDS/5 % β‐mercaptoethanol/0.01 % Bromophenol Blue), and incubated at 95 °C for 10 min. Whole cell lysates were resolved by 4–20 % sodium dodecylsulfate polyacylamide gel electrophoresis (SDS‐PAGE; 200 V for 25 min) followed by electro transfer to polyvinylidene difluoride membrane, PVDF (350 mA for 1 h). Membranes were blocked in 5 % (w/v) non‐fat milk in PBST (PBS/0.1 % Tween 20) and incubated with the appropriate primary antibodies (Cell Signalling Technology). After incubation with horseradish peroxidase‐conjugated secondary antibodies (Cell Signalling Technology), immune complexes were detected with the ECL detection reagent (Bio‐Rad) and analysed using a chemiluminescence imager (Bio‐Rad ChemiDoc Imaging System).

**Annexin V‐propidium iodide assay**: U2OS cells were seeded in 6‐well plates (at a density of 5×10^5^ cells/mL) and the cells were allowed to attach overnight. The cells were incubated without and with **1** (IC_50_ value×2 for 72 h) or cisplatin (12.5 μM for 72 h) at 37 °C. Cells were then harvested by trypsinisation. The FITC Annexin V/Dead Cell Apoptosis Kit was used. The manufacturer's (Thermo Fisher Scientific) protocol was followed to carry out this experiment. Briefly, harvested untreated and treated cells (1×10^6^) were suspended in 1×Annexin binding buffer (100 μL) (10 mM HEPES, 140 mM NaCl, 2.5 mM CaCl_2_, pH 7.4), then 5 μL FITC Annexin V and 1 μL PI (100 μg/mL) were added to each sample and incubated at room temperature for 15 min. After which, more 1×Annexin binding buffer (400 μL) was added while gently mixing. The cells were analysed using a FACSCanto II flow cytometer (BD Biosciences) (10,000 events per sample were acquired) at the University of Leicester FACS Facility. The FL1 channel was used to assess Annexin V binding and the FL2 channel was used to assess PI uptake. Cell populations were analysed using the FlowJo software (Tree Star).

**COX‐2 expression assay**: U2OS‐MTX cells were seeded in 6‐well plates (at a density of 5×10^5^ cells/mL) and the cells were allowed to attach overnight. The cells were treated with lipopolysaccharide (LPS) (2.5 μg/mL for 24 h), and then treated with **1** (IC_50_ value and IC_50_ value×2) or salicylic acid (20 μM) and incubated for a further 48 h. The cells were then harvested by trypsinization, fixed with 4 % paraformaldehyde (at 37 °C for 10 min), permeabilised with ice‐cold methanol (for 30 min), and suspended in PBS (200 μL). The Alexa Fluor® 488 nm labelled anti‐COX‐2 antibody (5 μL) was then added to the cell suspension and incubated in the dark for 1 h. The cells were then washed with PBS (1 mL) and analysed using a FACSCanto II flow cytometer (BD Biosciences) (10,000 events per sample were acquired) at the University of Leicester FACS Facility. The FL1 channel was used to assess COX‐2 expression. Cell populations were analysed using the FlowJo software (Tree Star).

## Conflict of interest

The authors declare no conflict of interest.

1

## Supporting information

As a service to our authors and readers, this journal provides supporting information supplied by the authors. Such materials are peer reviewed and may be re‐organized for online delivery, but are not copy‐edited or typeset. Technical support issues arising from supporting information (other than missing files) should be addressed to the authors.

Supporting Information

## Data Availability

The data that support the findings of this study are available from the corresponding author upon reasonable request.
