# Supplementary material for: The Osteosarcoma Stem Cell Activity of a Gallium(III)‐Phenanthroline Complex Appended to Salicylate
Source: Chembiochem. 2022 Nov 18;23(24):e202200532. doi: 10.1002/cbic.202200532 (PMC10099568; doi:10.1002/cbic.202200532)
Supplement: Supplementary file 1 — Supporting Information [file CBIC-23-0-s001.pdf]

ChemBioChem

Supporting Information

The Osteosarcoma Stem Cell Activity of a Gallium(III)-Phenanthroline Complex Appended to Salicylate

Ruby A. Vincent[+], Ginevra Passeri[+], Joshua Northcote-Smith, Kuldip Singh, and Kogularamanan Suntharalingam*

Table of Content

Figure S1. ^1H NMR spectrum of **1** in CD_3CN.
Figure S2. $^{31}P\{^1H\}$ NMR spectrum of **1** in CD_3CN.
Figure S3. $^{19}F\{^1H\}$ NMR spectrum of **1** in CD_3CN.
Figure S4. ATR-FTIR spectrum of **1** in the solid form.
Figure S5. ESI mass spectrum (positive mode) of **1**.
Figure S6. ^1H NMR spectrum of salicylic acid in CD_3CN.
Table S1. Crystallographic data for complex **1**.

Table S2. Selected bond lengths (Å) and angles (°) for complex **1**.
Table S3. Experimentally determined LogP values for **1** and **2**.
Figure S7. UV-Vis spectrum of **1** (50 μM) in DMSO over the course of 24 h at 37 ^oC.
Figure S8. UV-Vis spectrum of **1** (50 μM) in H_2O:DMSO (200:1) over the course of 24 h at 37 ^oC.
Figure S9. UV-Vis spectrum of **1** (50 μM) in PBS:DMSO (200:1) over the course of 24 h at 37 ^oC.
Figure S10. UV-Vis spectrum of **1** (50 μM) in the presence of ascorbic acid (500 μM) in PBS:DMSO (200:1) before and after incubation for 24 h at 37 ^oC.
Figure S11. UV-Vis spectrum of **1** (50 μM) in DMEM:DMSO (200:1) over the course of 24 h at 37 ^oC.
Figure S12. Representative dose-response curves for the treatment of U2OS or U2OS-MTX cells with **1** after 72 h incubation.
Figure S13. Representative dose-response curves for the treatment of U2OS or U2OS-MTX cells with **2** after 72 h incubation.
Figure S14. Representative dose-response curves for the treatment of U2OS or U2OS-MTX cells with salicylic acid after 72 h incubation.
Figure S15. Representative dose-response curves for the treatment of MCF10A cells with **1** after 72 h incubation.
Figure S16. Representative bright-field images (× 10) of U2OS-MTX sarcospheres in the absence and presence of cisplatin, doxorubicin, or salinomycin at their respective IC_{20} values for 10 days.
Figure S17. Representative dose-response curves for the treatment of U2OS-MTX sarcospheres with **1**, **2**, or salicylic acid after 10 days incubation.
Figure S18. Representative dose-response curves for the treatment of U2OS-MTX cells with **1** in the presence of z-VAD-FMK (5 μM) after 72 h incubation.
Figure S19. Representative histograms displaying the green fluorescence emitted by anti-COX-2 Alexa Fluor 488 nm antibody-stained U2OS-MTX cells treated with LPS (2.5 μg/mL) for 24 h followed by 48 h in fresh media (red) or media containing salicylic acid (20 μM, blue).
Figure S20. Representative dose-response curves for the treatment of U2OS-MTX cells with **1** in the presence of PGE2 (20 μM) after 72 h incubation.

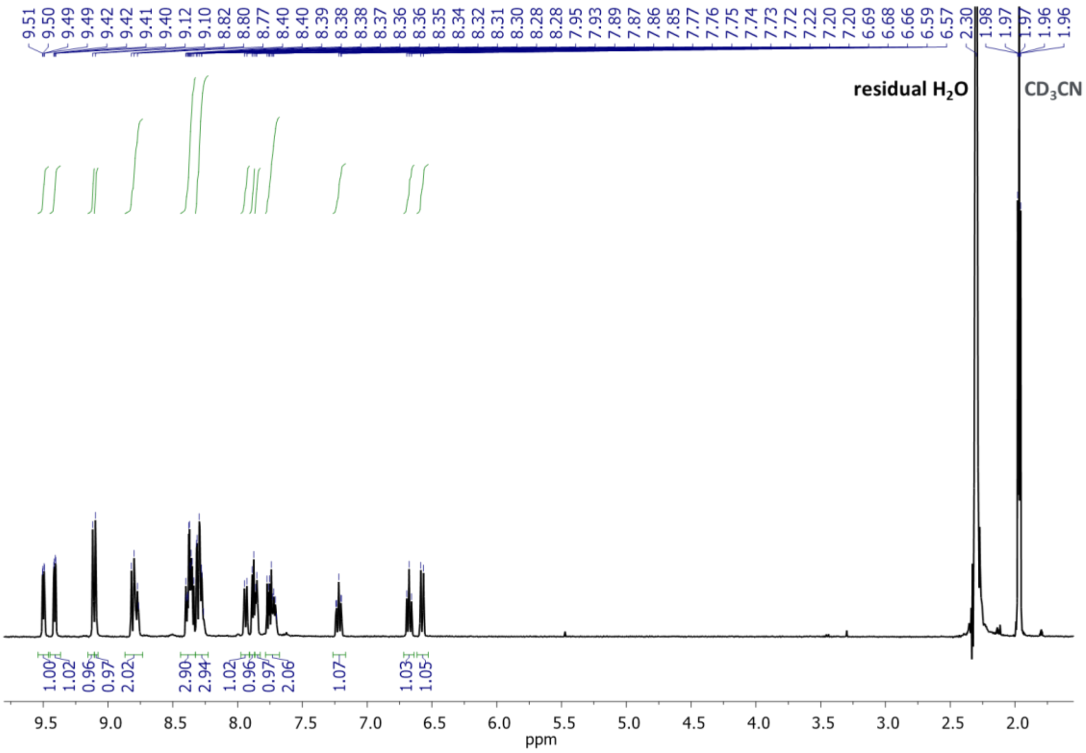

Figure S1. ^{1}H NMR spectrum of **1** in CD_3CN.

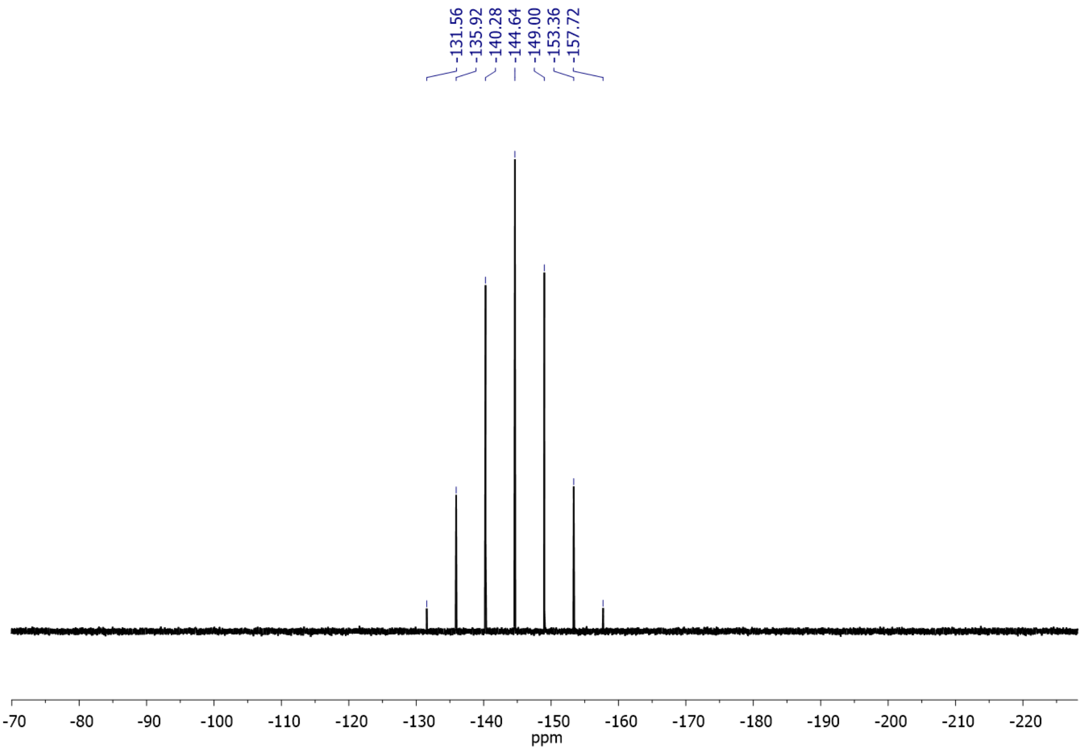

Figure S2. $^{31}P\{^1H\}$ NMR spectrum of **1** in CD_3CN.

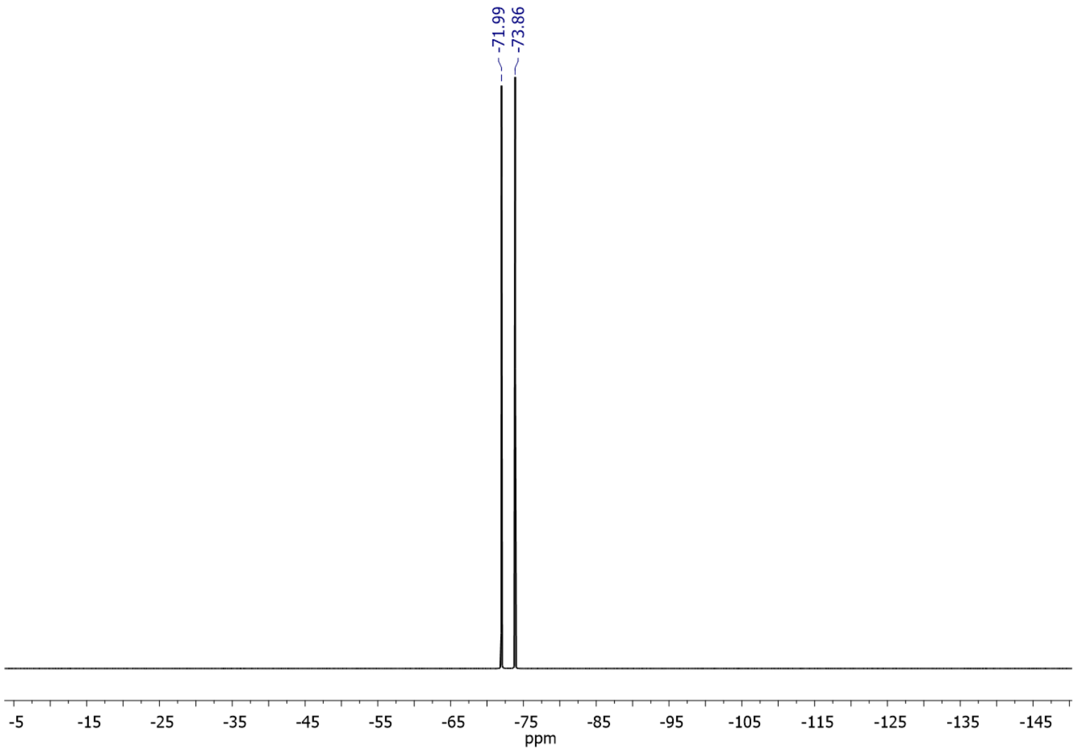

Figure S3. $^{19}F\{^{1}H\}$ NMR spectrum of **1** in CD_3CN.

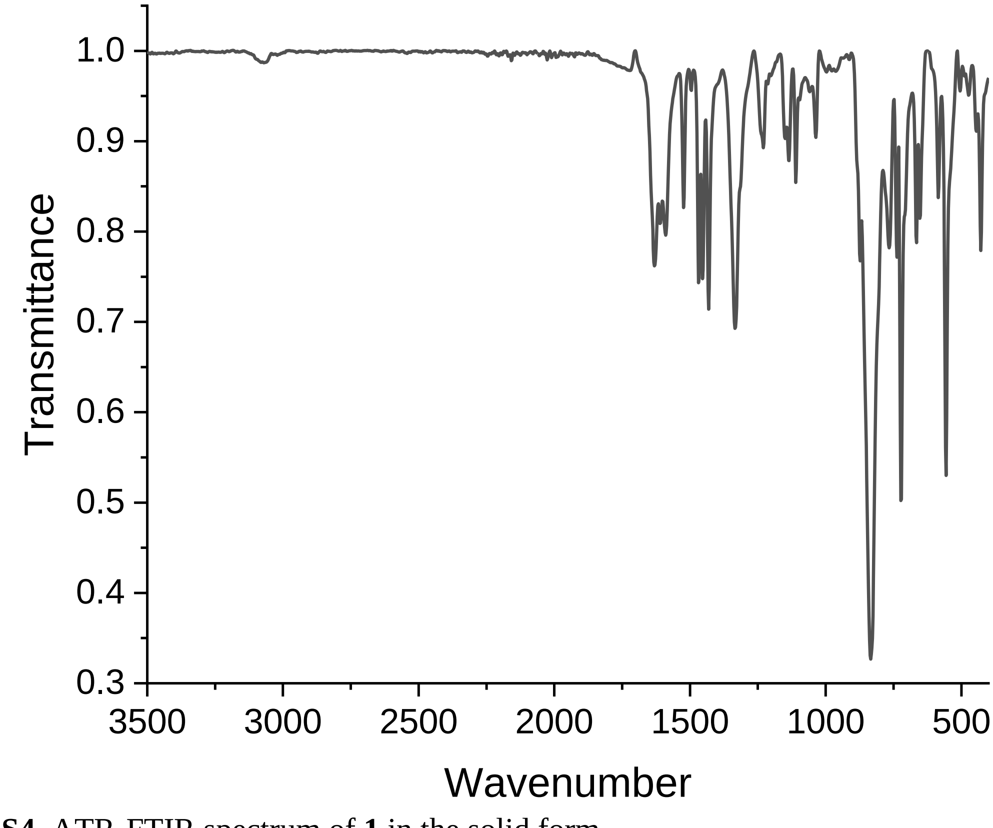

Figure S4. ATR-FTIR spectrum of **1** in the solid form.

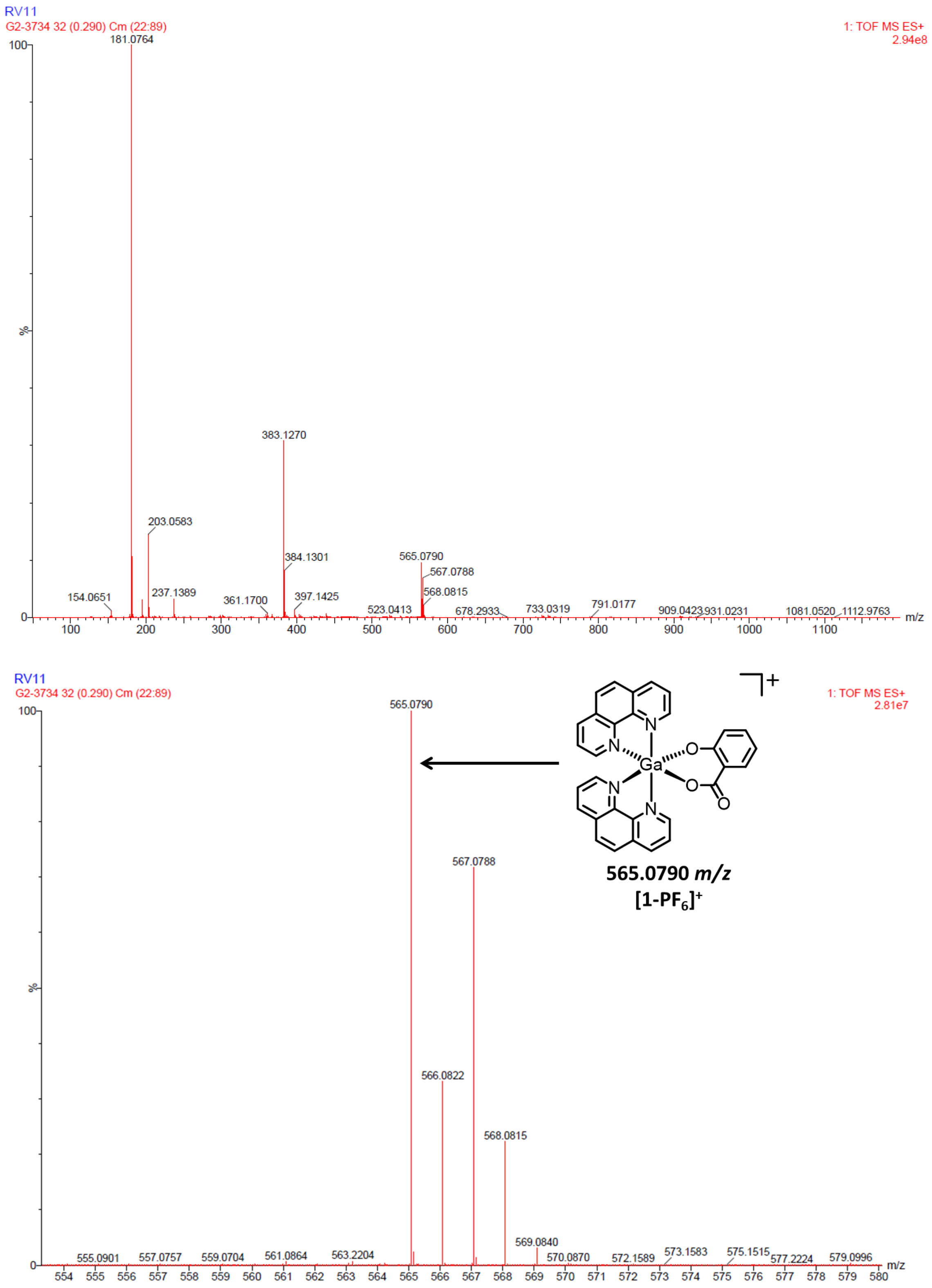

Figure S5. ESI mass spectrum (positive mode) of **1**.

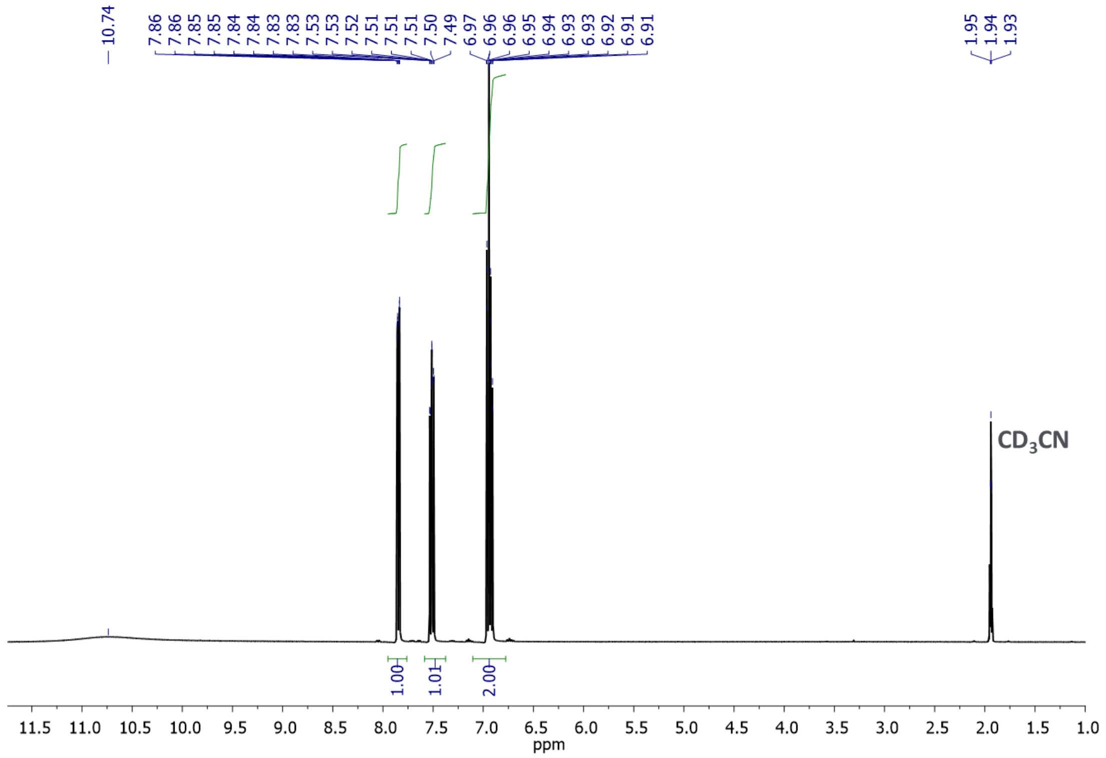

Figure S6. ^{1}H NMR spectrum of salicylic acid in CD_3CN.

Table S1. Crystallographic data for complex **1**.

**Metal complex**	**1**
CCDC No.	2205005
formula	$C_{31}H_{20}F_6GaN_4O_3P \cdot CH_3CN$
Fw	752.25
Crystal system	monoclinic
Space group	$P2_1/c$
a, Å	15.9081(6)
b, Å	11.7043(5)
c, Å	16.6318(6)
α, deg.	90
β, deg.	101.449(2)
γ, deg.	90
V, $Å^3$	3035.1(2)
Z	4
D_{calcd}, Mg/m^3	1.646
2θ / deg.	5.668 to 144.62
Reflections collected	52500
Independent reflections	5978 [R_{int} = 0.0694, R_{sigma} = 0.0389]
Goodness-of-fit on F^2	1.037
R_1, wR_2 [$I \geq 2\sigma(I)$]	R_1 = 0.0604, wR_2 = 0.1654
R_1, wR_2 [all data]	R_1 = 0.0642, wR_2 = 0.1733
Largest diff. peak/hole / e $Å^{-3}$	1.90/-1.50

Table S2. Selected bond lengths (Å) and angles (°) for complex **1**.

Ga(1)-O(1)	1.9032(19)	Ga(1)-N(2)	2.094(2)
Ga(1)-O(2)	1.8733(17)	Ga(1)-N(3)	2.074(2)
Ga(1)-N(1)	2.088(2)	Ga(1)-N(4)	2.141(2)
O(1)-Ga(1)-N(1)	90.44(8)	O(2)-Ga(1)-N(4)	169.49(8)
O(1)-Ga(1)-N(2)	169.39(9)	N(1)-Ga(1)-N(2)	78.95(9)
O(1)-Ga(1)-N(3)	94.75(8)	N(1)-Ga(1)-N(4)	88.21(9)
O(1)-Ga(1)-N(4)	90.36(8)	N(2)-Ga(1)-N(4)	89.47(8)
O(2)-Ga(1)-O(1)	95.91(8)	N(3)-Ga(1)-N(1)	165.26(9)
O(2)-Ga(1)-N(1)	100.14(9)	N(3)-Ga(1)-N(2)	95.59(8)
O(2)-Ga(1)-N(2)	85.92(8)	N(3)-Ga(1)-N(4)	77.99(9)
O(2)-Ga(1)-N(3)	93.04(8)		

Table S3. Experimentally determined LogP values for **1** and **2**.

Gallium(III) complex	LogP
**1**	0.27 ± 0.01
**2**	-0.48 ± 0.01

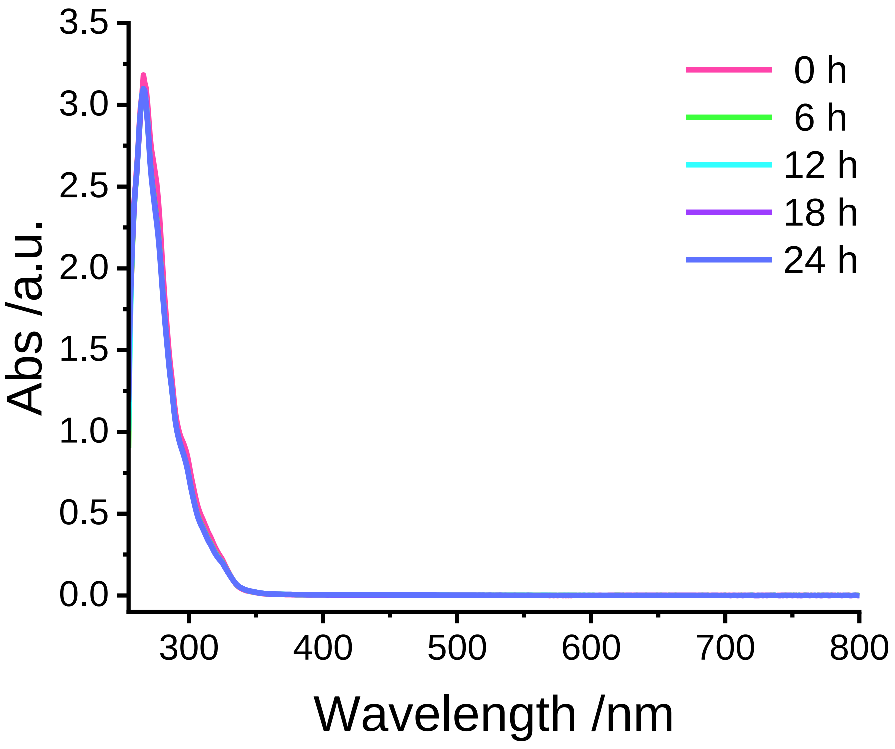

Figure S7. UV-Vis spectrum of **1** (50 μM) in DMSO over the course of 24 h at 37 °C.

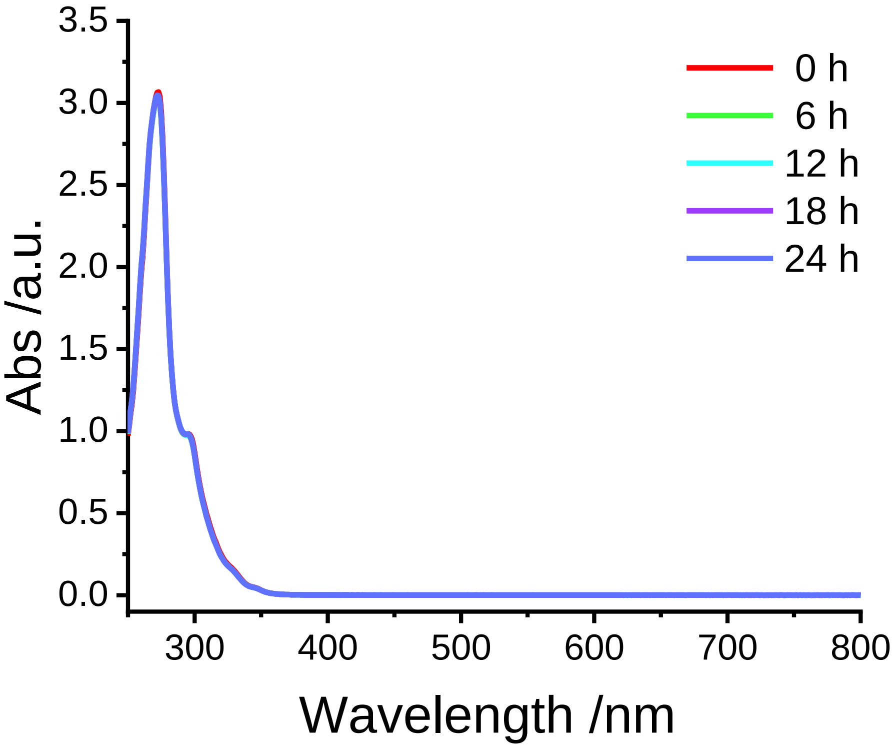

Figure S8. UV-Vis spectrum of **1** (50 μM) in H_2O:DMSO (200:1) over the course of 24 h at 37 °C.

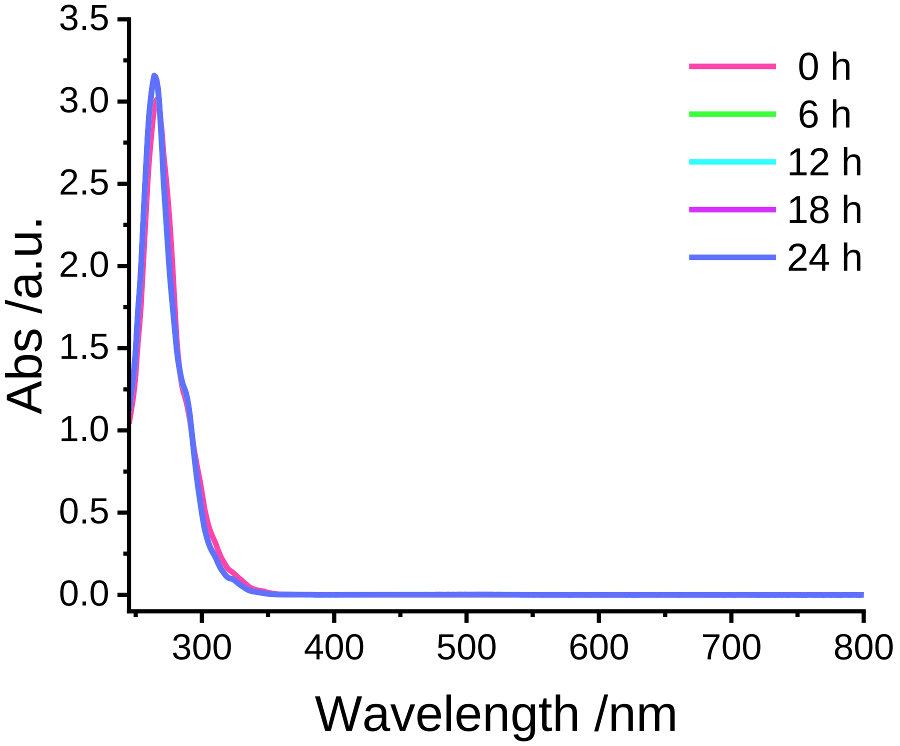

Figure S9. UV-Vis spectrum of **1** (50 µM) in PBS:DMSO (200:1) over the course of 24 h at 37 ^{o}C.

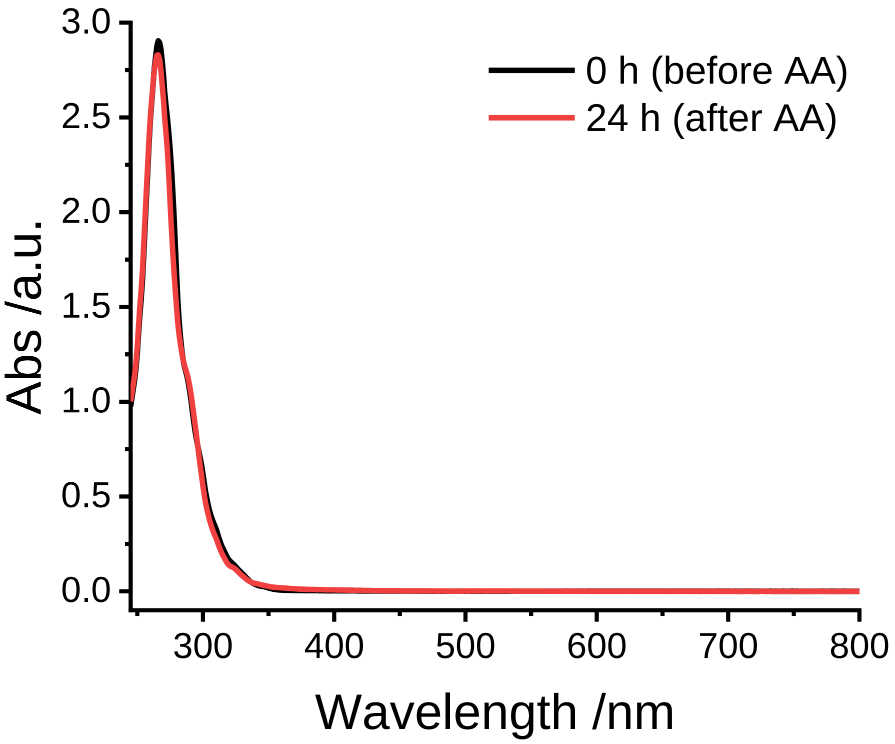

Figure S10. UV-Vis spectrum of **1** (50 µM) in the presence of ascorbic acid (500 µM) in PBS:DMSO (200:1) before and after incubation for 24 h at 37 ^{o}C.

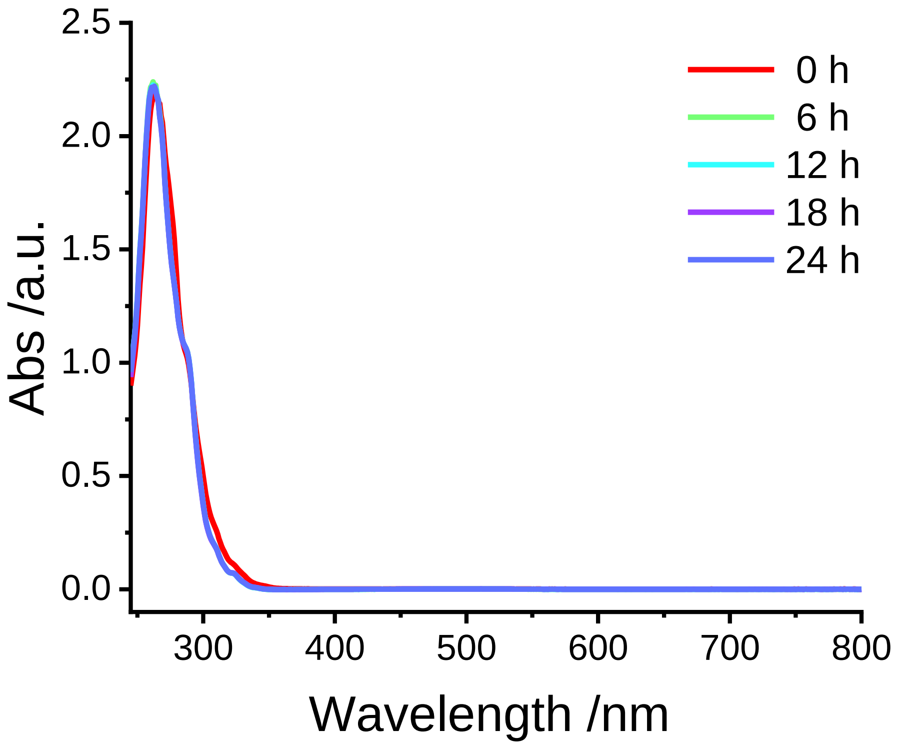

Figure S11. UV-Vis spectrum of **1** (50 μM) in DMEM:DMSO (200:1) over the course of 24 h at 37 °C.

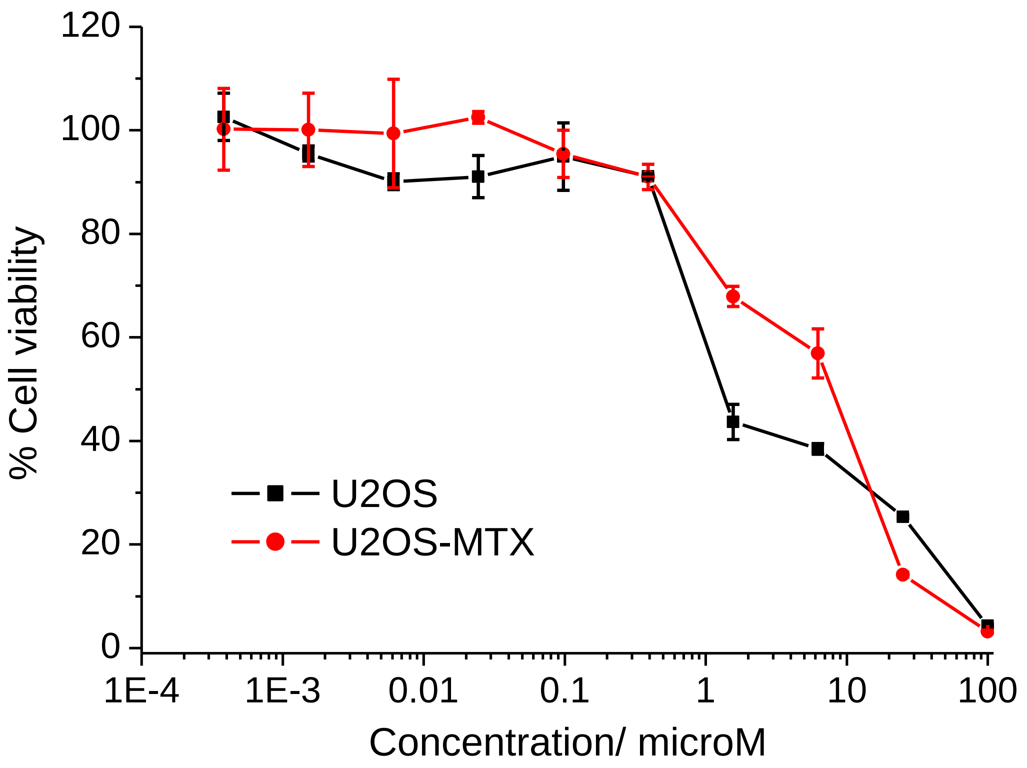

Figure S12. Representative dose-response curves for the treatment of U2OS or U2OS-MTX cells with **1** after 72 h incubation.

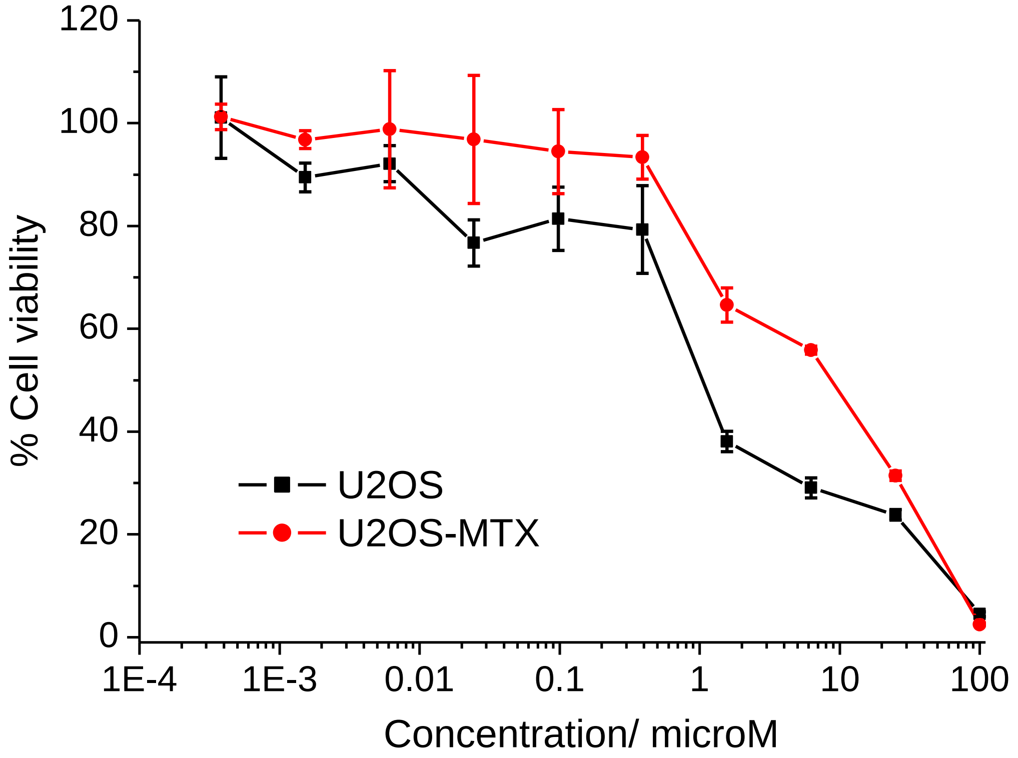

Figure S13. Representative dose-response curves for the treatment of U2OS or U2OS-MTX cells with **2** after 72 h incubation.

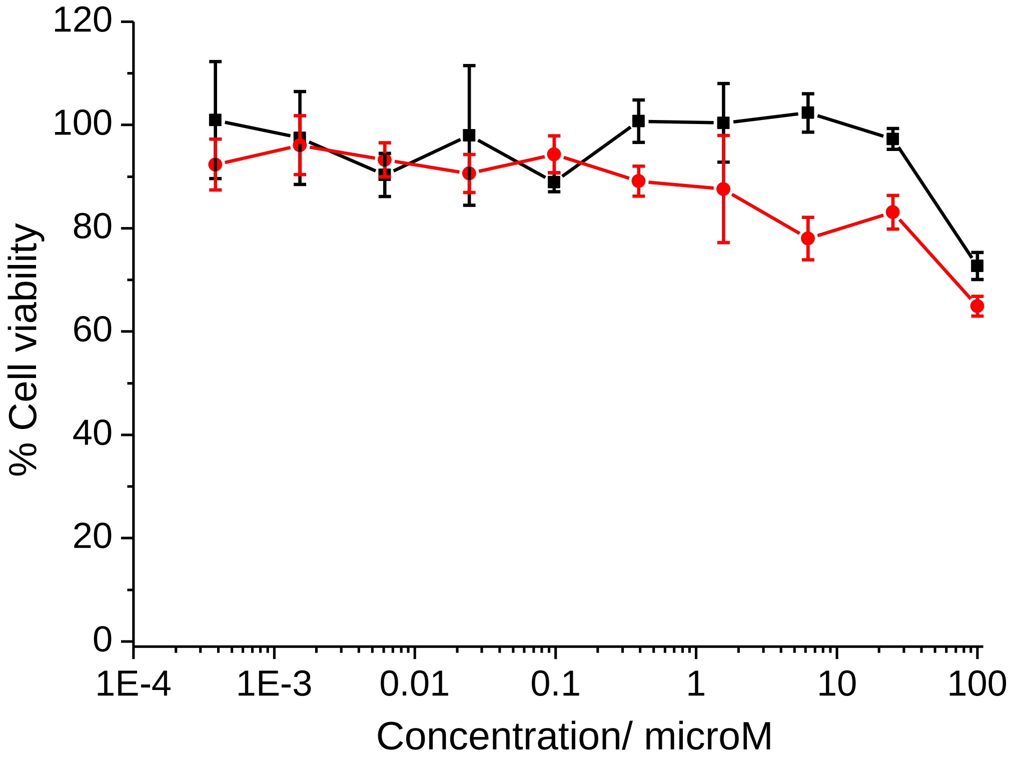

Figure S14. Representative dose-response curves for the treatment of U2OS or U2OS-MTX cells with salicylic acid after 72 h incubation.

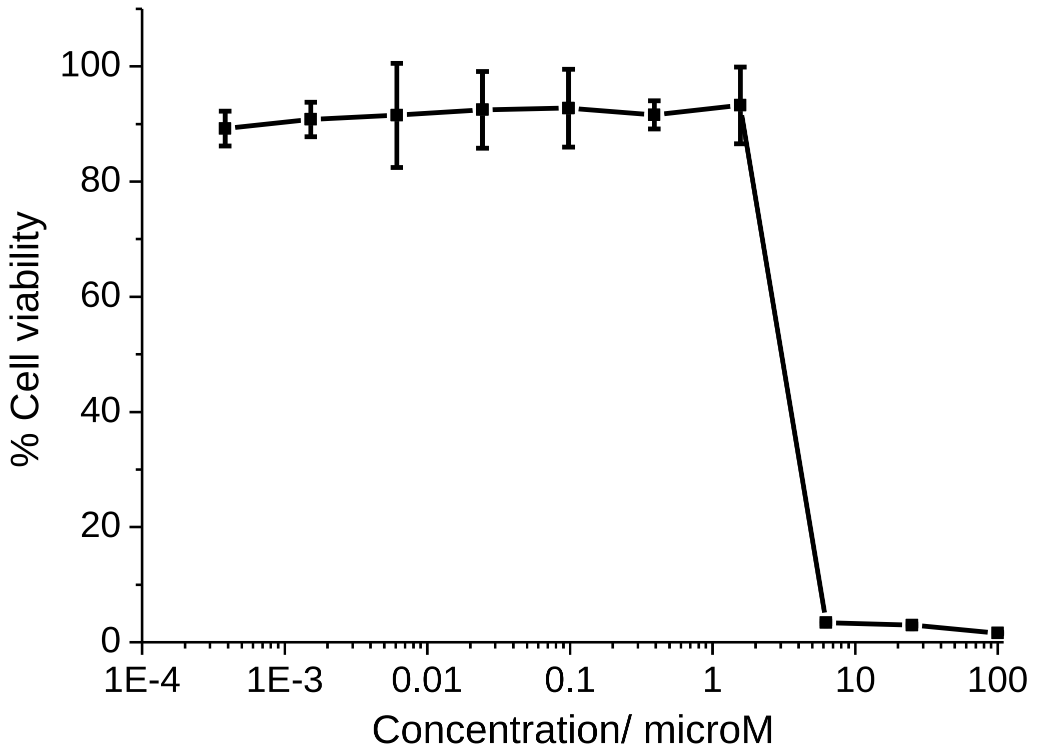

Figure S15. Representative dose-response curves for the treatment of MCF10A cells with **1** after 72 h incubation.

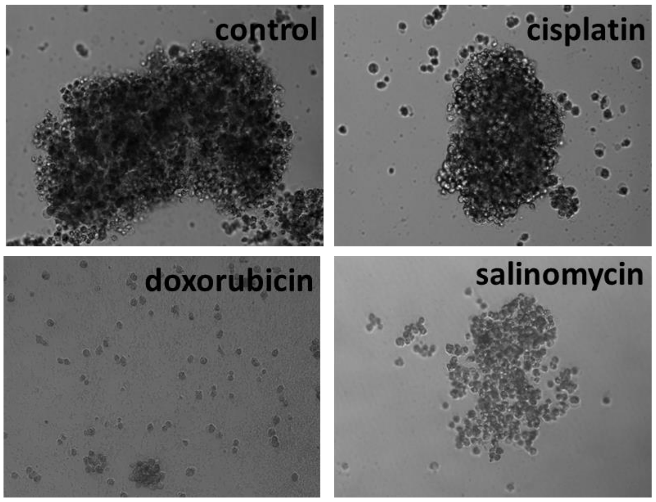

Figure S16. Representative bright-field images (× 10) of U2OS-MTX sarcospheres in the absence and presence of cisplatin, doxorubicin, or salinomycin at their respective IC_{20} values for 10 days.

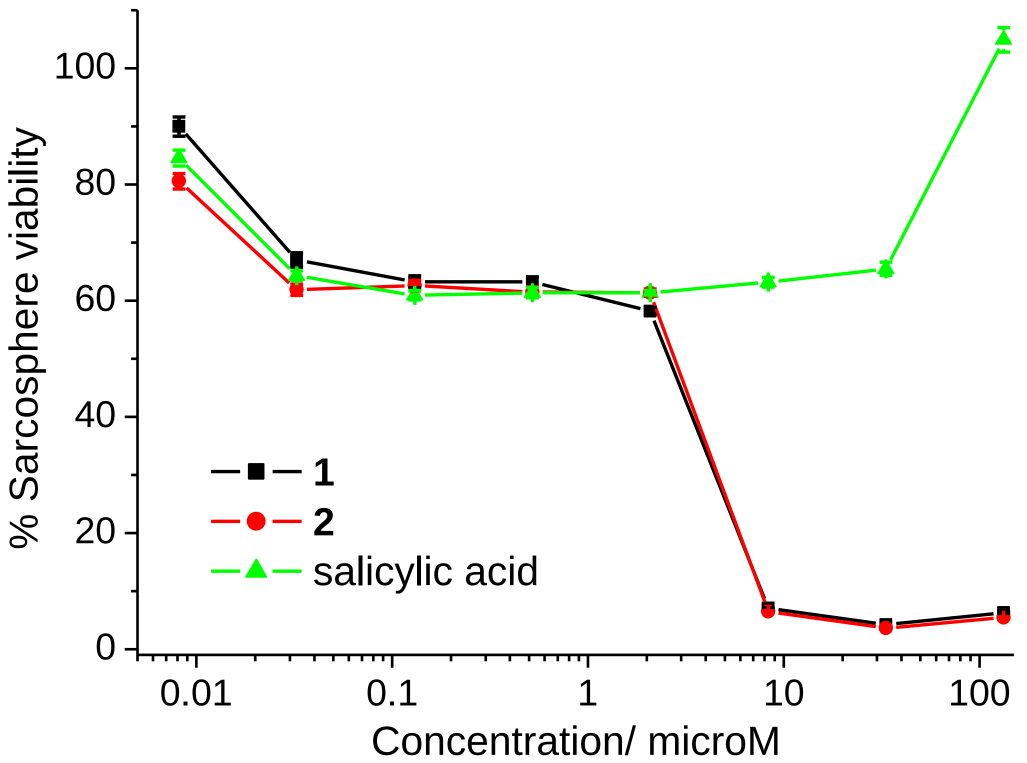

Figure S17. Representative dose-response curves for the treatment of U2OS-MTX sarcospheres with **1**, **2**, or salicylic acid after 10 days incubation.

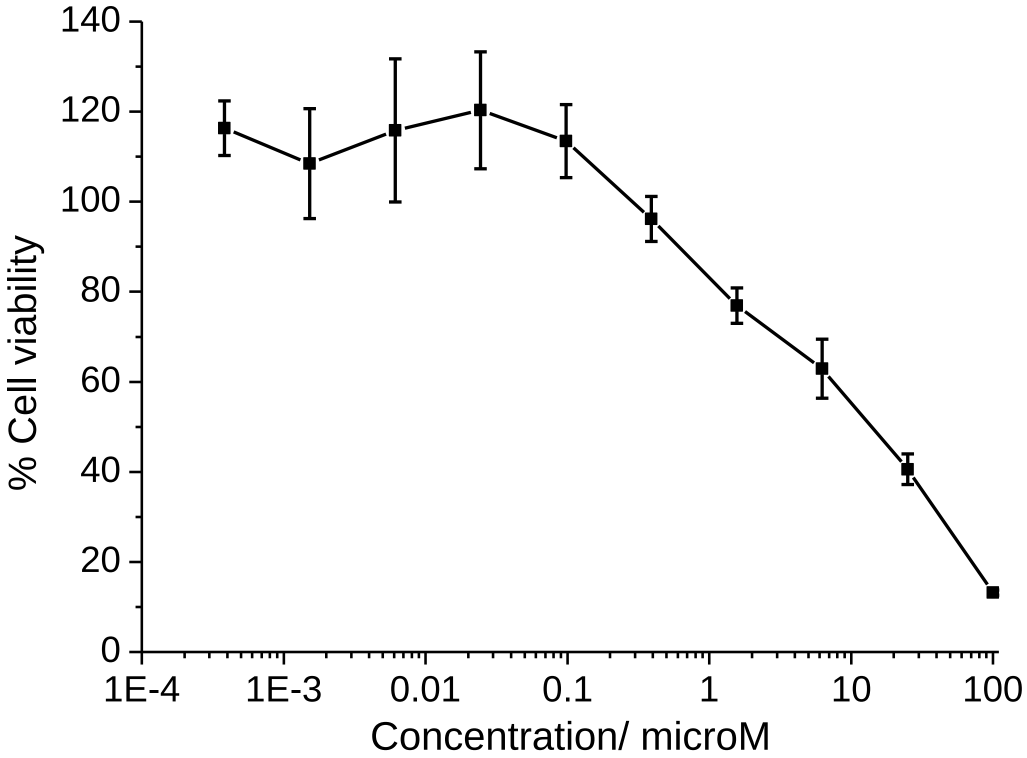

Figure S18. Representative dose-response curves for the treatment of U2OS-MTX cells with **1** in the presence of z-VAD-FMK (5 µM) after 72 h incubation.

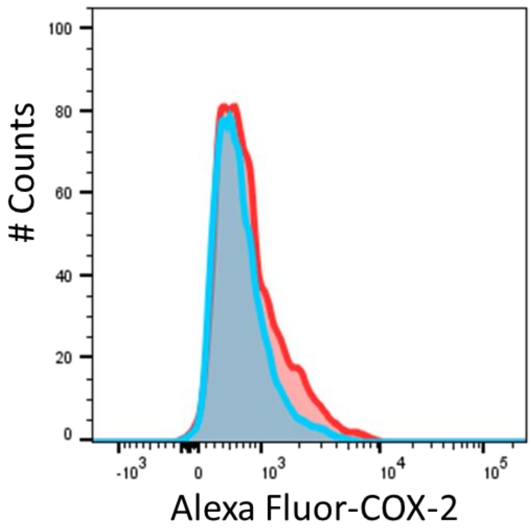

Figure S19. Representative histograms displaying the green fluorescence emitted by anti-COX-2 Alexa Fluor 488 nm antibody-stained U2OS-MTX cells treated with LPS (2.5 µg/mL) for 24 h followed by 48 h in fresh media (red) or media containing salicylic acid (20 µM, blue).

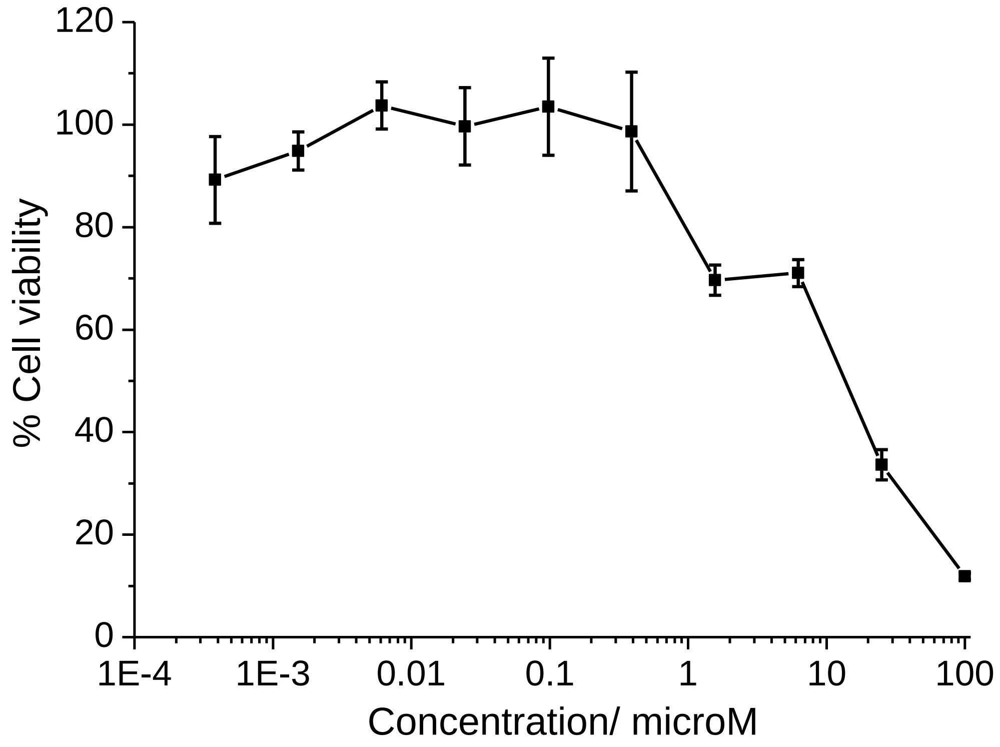

Figure S20. Representative dose-response curves for the treatment of U2OS-MTX cells with **1** in the presence of PGE2 (20 µM) after 72 h incubation.
